# Psychedelics as moral bioenhancers: Protocol for a scoping review of ethical arguments for and against

**DOI:** 10.12688/wellcomeopenres.23414.1

**Published:** 2025-01-13

**Authors:** Samuel Streicher, Christopher Register, Xiu Lim, Maide Barış, Sebastian Porsdam Mann, Katherine Cheung, Emma C Gordon, David Yaden, Julian Savulescu, Brian D. Earp

**Affiliations:** 1 University of Ottawa Faculty of Medicine, Ottawa, Ontario, Canada; 2 University of Oxford Uehiro Centre for Practical Ethics, Oxford, UK; 3 Choate Rosemary Hall, Wallingford, Connecticut, USA; 4 Marmara University School of Medicine, Istanbul, Turkey; 5 Centre for Advanced Studies in Bioscience Innovation Law, Faculty of Law, University of Copenhagen, Copenhagen, Denmark; 6 University of Oxford Faculty of Law, Oxford, UK; 7 Johns Hopkins University Berman Institute of Bioethics, Baltimore, Maryland, USA; 8 Department of Philosophy, University of Glasgow, Glasgow, UK; 9 Center for Psychedelic and Consciousness Research, Johns Hopkins University School of Medicine, Baltimore, Maryland, USA; 10 National University Singapore Centre for Biomedical Ethics,Yong Loo Lin School of Medicine, Singapore, Singapore

**Keywords:** Psychedelic, hallucinogen, moral enhancement, bioenhancement, scoping review, protocol

## Abstract

**Background:**

Moral bioenhancement typically refers to the deliberate use of drugs or biotechnologies, potentially alongside other practices, to attempt to improve oneself morally. In addition to general concerns regarding moral self-bioenhancement, the possibility of using psychedelic substances for such purposes raises distinct ethical questions. As a first step in analysing these questions, we intend to perform a scoping review of the existing arguments for and against the use of psychedelics as moral bioenhancers. We will focus primarily on voluntary use by individuals, although voluntary use by couples or small groups will be considered. The present contribution is a protocol for this scoping review.

**Methods:**

Our scoping review will adhere to the Joanna Briggs Institute methodology, which involves five stages: (1) identifying the research question, (2) developing the search strategy, (3) setting inclusion criteria, (4) extracting data, and (5) presenting and analysing the results. We will include both published and unpublished sources if they explicitly present ethical arguments for or against the voluntary use of psychedelics as intentional moral bioenhancers in adults. We will search for relevant studies in Embase, MEDLINE, Web of Science, Google Scholar, The National Library of Medicine, the Cumulated Index to Nursing and Allied Health Literature, Philosopher’s Index, the Bioethics Literature Database, EthxWeb, PhilPapers, Stanford Encyclopedia of Philosophy, Philosopher’s Index, EBSCO, BASE, and WorldCat. Sources will be excluded if (a) the full text is inaccessible, (b) the main text is in a language other than English, or (c) the focus is not primarily on ethical arguments (for example, focusing primarily on the clinical use of psychedelics for treatment). Two raters will independently assess all articles for eligibility, with disagreements to be resolved with a third reviewer. Data from eligible articles will be charted using a standardised data extraction form. The data will be analysed following PRISMA-ScR guidelines.

## Plain Language Summary

Moral bioenhancement typically refers to the deliberate use of drugs or biotechnologies, potentially alongside other practices, to attempt to improve oneself morally. In addition to general concerns regarding moral enhancement, the possibility of using psychedelic substances for such purposes raises distinct ethical questions. As a first step in analysing these questions, we intend to perform a scoping review of the existing arguments for and against the use of psychedelics as moral bioenhancers. We will focus primarily on voluntary use by individuals, although voluntary use by couples or small groups will be considered. The present contribution is a protocol for this scoping review.

We will include both published and unpublished sources if they explicitly present ethical arguments for or against the voluntary use of psychedelics as intentional moral bioenhancers in adults. We will search for relevant studies in Embase, MEDLINE, Web of Science, Google Scholar, The National Library of Medicine, the Cumulated Index to Nursing and Allied Health Literature, Philosopher’s Index, the Bioethics Literature Database, EthxWeb, PhilPapers, Stanford Encyclopedia of Philosophy, Philosopher’s Index, EBSCO, BASE, and WorldCat. Sources will be excluded if (a) the full text is inaccessible, (b) the main text is in a language other than English, or (c) the focus is not primarily on ethical arguments (for example, focusing primarily on the clinical use of psychedelics for treatment). Two raters will independently assess all articles for eligibility, with disagreements to be resolved with a third reviewer. Data from eligible articles will be charted using a standardised data extraction form.

## Introduction

### Moral bioenhancement, psychedelics & research objectives

Ethical debates surrounding human enhancement often focus on cognitive or physical abilities. Critics argue that using certain technologies or substances to improve such capacities beyond what is necessary for medical treatment is ethically dubious, and may, moreover, amount to cheating or other wrongdoing, for example in certain competitive settings ( Porsdam Mann *et al.*, 2018; Schermer, 2008). However, the idea that biomedical enhancement is always or necessarily morally problematic was challenged in an influential paper by Douglas (2008), who introduced the concept of *moral enhancement* (also sometimes termed moral *bioenhancement* or - *neuroenhancement*). ^1^ Douglas (2008) argued that if we could use biotechnology to make ourselves *more moral*, it would be counterintuitive to consider this use of biotechnology as unethical (e.g., unfair, harmful, or disrespectful to others). After all, becoming more moral -- by whatever means -- seems to imply treating others better or adhering more closely to ethical principles. This argument thus turned traditional moral objections to human enhancement on their head.

However, the notion of moral bioenhancement has been heavily criticised. Some philosophers point to a fundamental challenge in defining what constitutes moral improvement, given a lack of universal agreement about matters of right and wrong ( Beck, 2015). Others worry about societal implications: could a morally enhanced population raise the bar for what's considered praiseworthy behaviour ( Porsdam Mann *et al.*, 2023), inadvertently marginalising those who haven't undergone such enhancements ( Archer, 2016)? Additionally, some suggest that focusing on individual moral bioenhancement might divert attention from systemic issues that contribute to unethical behaviour in society ( Hardcastle, 2018; Melo-Martín & Salles, 2015). On the other hand, moral bioenhancement may promote the greater good, for example, by reducing prejudice or increasing generosity ( Crutchfield, 2021). Indeed, Persson and Savulescu (2008) have also argued that there is an urgent *imperative* for such enhancement, given that it is generally easier to cause harm than benefit; that our ability to cause significant (or even existential-level) harm through technology is increasing ( Law *et al.*, 2024); and that the technological world is advancing at a much greater pace relative to developments in human moral psychology.

Even if it were supposed that moral bioenhancement is desirable, and that significant conceptual disagreements around what counts as such could be resolved, its implementation in practice would not be straightforward. Assuming that a bio-intervention such as a drug could precisely strengthen certain traits or capacities commonly regarded as virtuous, such as empathy, or decrease other, potentially counter-moral traits, such as aggression, it would not follow that a user would be all-things-considered morally enhanced ( Azevedo, 2016; Melo-Martín & Salles, 2015). For example, an inability to objectively and appropriately address sexual or other abuse due to strengthened -- but misplaced -- empathy for abusers would be morally problematic ( Sparrow, 2014). Likewise, decreased aggression would not be morally desirable if it prevented someone from applying necessary and proportionate force to defend an innocent party from an attacker.

Attempting to "fine-tune" a person's moral traits or capacities through the use of drugs or other biotechnologies has thus been regarded as a potential dead end for moral bioenhancement by some authors ( Dubljević & Racine, 2017; Wiseman, 2014; Wiseman, 2016). ^2^ As an additional complication, interventions that would work at more general levels to better enable people to respond appropriately to moral reasons in flexible, context-sensitive ways might seem to reduce to 'traditional' means of moral education such as socialisation or, perhaps, the use of cognitive enhancement technologies to improve general reasoning ( Harris, 2011). And yet, as Sparrow (2017) has argued, if the debate on "moral enhancement" collapses into questions around traditional moral education or cognitive enhancement, we would be simply changing the conversation.

In response to such concerns, Earp (2018) laid out three desiderata for a moral bioenhancer properly so-called. It must:

1. keep the 'bio' in moral bioenhancement [note that moral neuroenhancement is a type of moral bioenhancement on this picture] (thus ruling out 'purely' social, environmental, or psycho-behavioural interventions);
2. avoid the pitfalls associated with low-level 'dial adjustment' (that is, attempts to biochemically intervene in, or fine-tune, particular cognitive or emotional capacities involved in moral decision-making, behaviour, and the like);
3. exert a more general or wide-ranging effect on the moral agent that would contribute to her moral improvement in a robust, sustainable, flexible-across-contexts sort of way, without simply collapsing into cognitive enhancement (p. 421).

Such criteria might well be disputed. For example, it could be argued that even relatively "blunt" interventions, such as the use of methylphenidate (e.g., Ritalin) for impulse control could count as a moral enhancer in certain cases, particularly when an individual's inability to control their behaviour is sufficiently problematic or liable to cause harm. Nevertheless, reflection on these criteria and the kind(s) of intervention that might fulfil them suggests a range of possibilities, one of which is psychedelics ( Gordon *et al.*, in press). The basis for this proposal is the observation that some human societies have, for millennia, used certain substances (i.e., the subset of plant medicines now commonly termed, within Western culture, *psychedelics* -- a relatively recent coinage that also applies to some synthetic drugs such as lysergic acid diethylamide or LSD), typically in conjunction with spiritual practices or other disciplines, to promote qualities that would widely be recognized as moral improvements. Accordingly, Earp (2018) suggested that these substances could, if used in the right way, potentially meet all three proposed desiderata and argued that they should therefore be considered more seriously in the moral bioenhancement debate.

Primarily stimulating neuronal serotonin (5-HT)2A receptors, so-called *classic* psychedelics temporarily reconfigure the typical connection patterns used by the brain for information processing, thus increasing neuroplasticity ( Carlsson *et al.*, 1999; Dean *et al.*, 2013; National Academies of Sciences, Engineering, & Medicine, 2022; Nichols, 2004; Nichols, 2016; Ling *et al.*, 2022; Lowe *et al.*, 2022). ^3^ This may allow some users to overcome habitual forms of thinking or responding that are harmful or maladaptive ( Vollenweider & Preller, 2020). Some users may then be better able to navigate multifaceted moral issues. Other reported effects that might seem to comport with the above criteria include: "increased patience, good-natured humour and playfulness, mental flexibility, optimism, interpersonal perceptiveness and caring, and compassion or social concern" ( Griffiths *et al.*, 2006, p. 280 as cited in Earp, 2018, pp. 430-431), "opening the heart, expanding love for others, and leading to healing of both self and relationships" ( Winkelman, 2015, p. 108), and "increas[ing] one's capacity to maintain perspective in response to strong emotional states" ( Soler *et al.*, 2016, as cited in Earp, 2018, p. 431).

Although adverse reactions to psychedelics can occur, they are often associated with factors that can be screened or mitigated prior to use, such as distressing environments, certain pre-existing psychiatric conditions, and improper dosing ( Móró *et al.*, 2011; Strassman, 1984, as cited in Earp, 2018). Classic psychedelics are generally regarded as non-addictive, and the doses required to effect altered states of consciousness are low or non-toxic ( Nichols, 2004). That being said, there are increasing calls to study the potential harms of psychedelics (and other substances often studied in conjunction with psychedelics, such as MDMA or ketamine), including harms associated with increased suggestibility and vulnerability to abuse (e.g., by unscrupulous therapists, 'healers,' or charismatic leaders) ( Ehrenkranz *et al.*, 2024; McNamee *et al.*, 2023; Villeneuve & Prescott, 2022).

Since the publication of Earp's paper, contributions to the bioethics literature focusing specifically on psychedelics as potential moral bioenhancers have multiplied. Examples include Gordon's (2022) work on facilitating dimensions of interpersonal trust likely to maximise the benefits of psychedelic moral enhancement; Wu's (2023) discussion of the entanglement between moral, cognitive and emotional enhancement through psychedelics; Rakić's (2023) exploration of psilocybin, in particular, as a potential moral enhancer; Kirkham and Letheby's (2024) suggestion that psychedelics might help foster environmental virtues; and Langlitz *et al*.'s (2021) proposal that the psychedelic moral enhancement debate ought to draw from history and anthropology. However, to date, there has been no systematic, comprehensive attempt to synthesise the existing arguments for and against the use of psychedelics as moral bioenhancers. Now that there are rapid policy changes toward greater drug access—including for non-medical purposes—in some jurisdictions, such as the US state of Oregon ( Siegel *et al.*, 2023; see also Sandbrink *et al.*, 2024), it is timely and important to bring together all existing work on this topic to inform and enrich academic and public policy debates. Accordingly, we propose to undertake a scoping review of the literature on psychedelic moral bioenhancement.

### Use of scoping review methodology

Scoping reviews identify and clarify key ideas within a field while also searching for knowledge gaps ( Munn *et al.*, 2018). Systematic reviews, on the other hand, frequently aim to draw broad, evidence-based conclusions; these multi-sourced conclusions are often used to determine best practices in clinical medicine ( Munn *et al.*, 2018; Tricco *et al.*, 2018). Although distinct, both types of reviews involve a rigorous examination of a given literature using a recorded, reproducible search method. In this way, both reviews tend to include the following components: a) comprehensive searches for relevant materials, b) whose results are screened against particular exclusion criteria, c) with these results subsequently aggregated ( Birchley & Ives, 2022; Grant & Booth, 2009; Page *et al.*, 2021; Tricco *et al.*, 2018).

A scoping review, rather than a systematic review, was determined to be the ideal method for our discussion because we intend to collect the various ethical stances on the use of psychedelics as moral bioenhancers, without using this collection of arguments to construct a singular, overarching conclusion or directive. We also seek to identify knowledge gaps in the current discourse on this topic by collecting information from a variety of sources and potential stakeholders ( Peters *et al.*, 2020).

## Methods

### Step 1 - Formulating the research question

Arksey and O’Malley (2005) describe an iterative process involving five main steps: identifying the research question; identifying relevant studies; study selection; charting the data; and collating, summarizing and reporting results. Partly inspired by Arksey and O'Malley (2005), we formulated the main research question of our scoping review: What are the ethical arguments related to the voluntary use of psychedelics as intentional moral bioenhancers among adults? ^4^

Moreover, several sub-questions emerged to guide the scoping review:

- Which populations -- and across which cultural, geographical, and historical contexts -- are assumed or observed to be using (or to have used) psychedelics for purposes of voluntary moral bioenhancement?
- Which specific substances are included in discussions about psychedelic moral enhancement (including associated substances such as MDMA or ketamine) and what are the proposed advantages or disadvantages of each with respect to the aim of moral bioenhancement?
- What are the main ethical reasons given for and against the voluntary use of psychedelics among adults for moral bioenhancement?
- What are the assumptions behind arguments surrounding the ethical status of such use (e.g., that such use should only be permitted if the drugs are legalised; that further research must be undertaken before any recommendations can be made, etc.)?
- What are the possible gaps in the ethical reasoning regarding the voluntary use of psychedelics among adults for moral bioenhancement?
- What is the quality of engagement with the scientific literature in ethical arguments about the voluntary use of psychedelics for moral bioenhancement in adults (e.g., selective citations; acknowledgment of critical views; superficial interpretations of evidence; over-emphasis of benefits relative to harms, and so on).

### Step 2 - Search strategy

In order to capture the above ideas within the scoping review, our search incorporates carefully conceived definitions for key terms.

***Defining Key Terms - Psychedelics.*** We draw on multiple sources to craft a broad definition of psychedelic, ensuring a comprehensive search strategy. Psychedelic drugs are usually categorised into one of the following three classes: tryptamines, phenethylamines, and lysergamides ( Lowe *et al.*, 2022). For the purpose of our scoping review, we define psychedelic as any psychoactive substance that falls within one of these drug classes, where the pharmacological mechanism of action considerably involves activation of the (5-HT)2A receptor ( Nichols, 2016). ^5^ This serotonergic qualifier excludes certain phenethylamines, such as amphetamines, that are used as stimulants rather than psychedelics and whose mechanism of action is not largely serotonergic ( Martin & Le, 2024; Sitte, 2023).

It is important to note that some substances present classification challenges. For instance, 3,4-Methylenedioxymethamphetamine (MDMA) may not clearly meet our defining criteria to be deemed *psychedelic* ( Earp, 2018; Nichols, 1986). MDMA causes a release of serotonin, dopamine, and norepinephrine to elicit its effects ( Check, 2004; McCann *et al.*, 1994; Nichols, 1986), but can also interact with the (5-HT)2A receptor ( Collins *et al.*, 2016; Nichols, 1986). Other psychedelic-like or hallucinogenic substances, such as ketamine and ibogaine, are also often discussed in concert with classic psychedelics, notwithstanding their different mechanisms of action ( "Ibogaine," n.d; Martin *et al.*, 1982). For the sake of comprehensiveness, especially given their frequent co-occurrence in discussions of psychedelics and some similarities in their practical effects, we have decided to include such grey-area substances in search terms, at least for initial article collection and analysis.

When describing how certain psychedelics are synthesised or isolated, the Alcohol and Drug Foundation (2024) mentions terms such as ergot fungus, magic mushrooms, Mexican peyote, San Pedro cactus, psilocin, and entactogen. Other research teams, including Lowe *et al.* (2022), also mention cultural and colloquial names for various psychedelic substances. Given the variety of psychedelic substances and their vast sociocultural history, we recognize that it would be impractical to attempt to include all cultural and colloquial names for psychedelic materials and derivatives. However, if a publication argues for or against the use of a psychedelic substance with a specific chemical, colloquial, or cultural name that we omitted from our search term list, we anticipate that the authors would have also used general terms - such as psychedelic, psychoactive, hallucinogen, and the like - to describe the nature or effect of this substance. If this idea is correct, the search terms provided below should be sufficiently broad to capture these types of publications.

***Defining Key Terms - Moral Enhancement.*** Scholars debate what counts as enhancement, especially when compared to what counts as treatment ( Zohny *et al.*, 2022; see also Savulescu *et al.*, 2011). We will adapt an existing definition of "moral (neuro)enhancement" as follows: "[a]ny change in a moral agent, A, effected or facilitated in some significant way by the application of a neurotechnology, that results, or is reasonably expected to result, in A's being a morally better agent" or behaving more morally under typical circumstances ( Earp *et al.*, 2017, p. 168). We require that an article explicitly use qualifiers such as *moral* or *ethical* to describe the form of enhancement involved. Articles that exclusively discuss enhancement outside the scope of moral improvement (e.g., cognitive enhancement only) will be screened out manually via our data extraction process, as described in the sections to follow.

***Defining key terms - arguments.*** We also exclude publications that merely describe the effects of psychedelics as moral bioenhancers without any position for or against their use. Indeed, our search requires that a publication provide an argument. By argument, we mean a stated or clearly implied bio/ethical and/or normative reason for or against the use of psychedelics as moral bioenhancers. Our decision to focus on bioethical arguments, specifically, rather than (a) primarily empirical, scientific claims or (b) broadly normative discussions connected to spirituality, religion, or Indigenous practice will help us avoid taking an overly wide lens.

Table 1 provides a search term summary according to the three fundamental terms above. Inspired by Strech & Sofaer (2012), we conceived of these terms through our initial review of the literature as encountered when drafting this protocol. Additionally, SS performed a cursory thesaurus search to broaden synonym inclusion. This cursory review was performed through Thesaurus.com. It is not necessary to include all possible synonyms, as publications that fall within our search parameters are likely to make use of at least one of the common terms listed in Table 1. Moreover, some databases allow users to search for any form of a word with a specified etymological root. For example, by using the search term enhance*, database X will interpret the asterisk as an instruction to search for any word that includes “enhance” within its spelling, such as enhancer, enhancement, and enhanced. For this reason, when practical, each term will be incorporated into the search thread by its root. The following keywords will also be adapted to relevant databases (e.g., MeSH terms in PubMed, Emtree terms in Embase) when possible.

**Table 1.** Fundamental Concepts and Associated Search Terms.

Fundamental Concept of Search Inquiry	Associated Search Terms
Psychedelic	Psychedelic, serotonergic, (5-HT)2A receptor, 5-hydroxytryptamine receptor 2A, hallucinogen, psychotomimetic, entheogen, empathogen, psychoactive, psychotropic, psychedelia, tryptamine, phenethylamine, lysergamide, psilocybin, psilocin, LSD, lysergic acid diethylamide, mescaline, DMT, dimethyltryptamine, 2C-B, 2-(4-bromo-2,5-dimethoxyphenyl)ethanamine, 4-Bromo-2,5-dimethoxyphenethylamine, peyote, MDMA, 3,4-Methylenedioxymethamphetamine, ecstasy, molly, mandy, entactogen, ergot, magic mushroom, kambo, ketamine, mescaline, San Pedro cactus, Lophophora williamsii, 25[-x]-NBOMe, N-methoxybenzyl, salvia, ayahuasca, bufo alvarius venom, huachuma, ibogaine
Moral	Moral, ethical, good, noble, principled, rational, honourable, insightful, proper, right, correct, courteous, decent, just, conscientious, virtuous
Enhancement	Enhance, bioenhance, neuroenhance, improve, augment, boost, build, heighten, increase, strengthen, amplify, upgrade, elevate, facilitate, benefit, therapy, transform
Argument	Argument, reason, claim, rationale, idea, motivation, logic, philosophy, judgement, mentality, sense, position, stance, good, bad, right, wrong, fair, immoral, unethical

Based on suggestions from BDE and CR, the key term *moral enhancement* has been split into its constituents to enable a more refined boolean search formula, provided below, given that this search focuses on moral enhancement in particular - not merely general enhancement.

***Boolean operators and formula.*** The Boolean operator AND will only be used to separate required search concepts, whereas the Boolean operator OR will be used for terms *within* each concept. It should be noted that we do not provide an overview of each database's unique instructions for keyword search here. Instead, each database's precise search thread will be included in the manuscript to follow. However, each database search thread will follow the approximate formula, with the expectation that some adaptations will be made to align with each database's search function:

*(Psychedelic Concept Term A OR Psychedelic Concept Term B OR Psychedelic Concept Term C . . .)*

AND

*(Moral Concept Term A OR Moral Concept Term B OR Moral Concept Term C . . .)*

AND

*(Enhancement Concept Term A OR Enhancement Concept Term B OR Enhancement Concept Term C . . .)*

AND

*(Argument Concept Term A OR Argument Concept Term B OR Argument Concept Term C . . .)*

***Database selection.*** To optimise our database selection, we consulted Bramer *et al*.'s (2017) study of systematic reviews. Although Bramer *et al.* (2017) analysed systematic reviews, their focus on database combinations’ recall ability is relevant for any project involving database searches, including scoping reviews. Bramer and colleagues analyse the references of 58 published systematic reviews to identify database combinations that produce high levels of reference recall (i.e., the number of included references retrieved by a specific database combination divided by the number of included references retrieved by all databases used by the respective publication), finding that "the combination of Embase, MEDLINE, Web of Science Core Collection, and Google Scholar performed best, achieving an overall recall of 98.3 and 100% recall in 72% of systematic reviews" (p. 245).

Following Bramer *et al*.'s (2017) recommendation to tailor database combinations to the project's specific field of research, we conducted additional research to identify bioethics-specific resources. To identify the most relevant bioethics-specific resources, SS performed a cursory Google search for popular bioethics databases. SS selected the first three university sites - Deakin University (2024), Georgetown University (2024), and Columbia University (2023) - to provide a list of databases. SS recorded any database, not already included in Bramer *et al*.'s work, that was listed by at least two of the three University sites, yielding the following recommendations: the National Library of Medicine (NLM), the Cumulated Index to Nursing and Allied Health Literature (CINAHL), Philosopher's Index, the Bioethics Literature Database (BELIT), and EthxWeb.

Informed by Grant and Booth (2009) as well as Barry *et al.* (2022), SS consulted Library and Information Science professionals to bolster the academic search. Dr. Hilla Wait, Philosophy and Theology Librarian at University of Oxford's Bodleian Libraries, suggested the following additional databases: PhilPapers, Stanford Encyclopedia of Philosophy, and Philosopher's Index.

We drew upon the diverse academic backgrounds of our research team, adding the following databases to the above collection: EBSCO, BASE, and WorldCat. Although BASE and WorldCat may be seen as our central grey literature sources, the following databases from our list also feature grey literature: Embase (Elsevier, 2024), Web of Science (Clarivate, 2023), Google Scholar ( Paez, 2017), CINAHL (Barry University, 2024), BELIT (University of Arkansas, 2017), EthxWeb (Georgetown Digital Repository, 2024), and PhilPapers (Philosophy Documentation Center, 2024).

Collectively, our database combination consists of Bramer *et al*.'s study conclusions plus the repeated database listings between Deakin, Georgetown, and Columbia University sites plus Dr. Wait's suggestions plus this research team's additions ( Table 2).

**Table 2.** Database Recommendations from Selected Authorities.

Expert Authority	Database Recommendations
Bramer *et al*.	• Embase • MEDLINE • Web of Science • Google Scholar
Repetitions among Deakin, Georgetown, and Columbia University	• The National Library of Medicine (NLM) • The Cumulated Index to Nursing and Allied Health Literature (CINAHL) • Philosopher’s Index • The Bioethics Literature Database (BELIT) • EthxWeb
Dr. Hilla Wait	• PhilPapers • Stanford Encyclopedia of Philosophy • Philosopher’s Index.
Research Team Additions:	• EBSCO • Grey literature sources: BASE and WorldCat

### Stage 3 - Inclusion and exclusion criteria

All identified citations will be compiled into Zotero, where duplicates will be removed. The remaining sources will then be imported into Covidence, a systematic review management software, for initial screening and review.

Included materials should focus on bioethical arguments or reasoning (concept) concerning the voluntary use of psychedelics as intentional moral bioenhancers (context) among adults (population). Our review will consider a range of English materials, including but not limited to published studies, systematic reviews, meta-analyses, editorials, book and thesis chapters, and policy statements. As noted earlier, sources that merely discuss the clinical use of psychedelics will be excluded.

Studies published or initiated from 2008 onward will be eligible for review. The starting date of 2008 was chosen because, to our knowledge, the specialised concept of "moral bioenhancement" was first introduced into the bioethics literature by Douglas in his 2008 work, "Moral Enhancement." In this work, Douglas presented a thorough discussion that specifically focused on moral enhancement through biomedical intervention, which initiated the ongoing debate on moral bioenhancement within the bioethics discipline ( Douglas, 2008).

Before beginning the data extraction process, SS and XL will independently test the inclusion and exclusion criteria on a random sample of 10% of the retrieved sources, categorising articles as 'Yes,' 'No,' or 'Maybe' based on titles and/or abstracts. The Kappa coefficient will be calculated to determine inter-rater reliability. If a Kappa value of 0.85 is not achieved during this initial screening, SS and XL will modify the inclusion and exclusion criteria; they will then screen a different 10% sample of the retrieved sources. This process will continue until a Kappa value of at least 0.85 is achieved ( McHugh, 2012).

Using the modified inclusion and exclusion criteria, SS and XL will categorise all articles as 'Yes,' 'No,' or 'Maybe' based on titles and/or abstract. All mutually agreed upon 'Yes' and/or 'Maybe' articles will proceed to full-text screening. Any disagreements ('Yes' vs 'No,' 'Maybe' vs 'No') will be resolved through discussion or by involving a third reviewer (BDE).

The full texts of remaining articles will be reviewed by SS and XL to make a final decision on their inclusion or exclusion.

The definitions and clarifications provided in the earlier subsection of this document will be considered when screening data at all stages. For instance, although we include terms such as "psychoactive" in Table 1, we will exclude from further review publications that discuss substances falling outside our definition of psychedelic. Indeed, we are not interested in just any psychoactive substance—such as caffeine and alcohol ( Alcohol and Drug Foundation, 2024; National Cancer Institute, 2011)—but are instead assessing *psychedelics* as moral bioenhancers.

### Stage 4 - Data extraction

Data will be extracted by SS & XL using Covidence online software (Veritas Health Innovation, Melbourne, Victoria, Australia). ^6^ Records for included sources will be maintained in case further verification is needed. We will use a standardised data extraction form - as outlined preliminarily in Table 3 - that will be iteratively updated as needed. Any disagreements between the reviewers (SS & XL) at this stage will also be resolved through discussion or by inviting an additional reviewer (BDE). If necessary, authors of the papers will be contacted to request missing or additional data.

**Table 3.** Standardised Data Extraction Form.

Category	Data to be Extracted
Article information	Author, journal/publication source, year of publication, DOI/URL, publication type (i.e., academic/scientific paper, grey literature, editorial, report, etc.), study/programme location
Aim of the study	What is the main purpose of the study or article? Is it to make an ethical argument, or to propose policy changes, etc.?
Definition (and the type) of psychedelics	How does the source describe/define psychedelics? Is there any specific type of psychedelic the source focuses on? How does this focused psychedelic substance allow for moral enhancement?
Definition of (moral) (bio)enhancement	How does the source describe/define (moral) (bio)enhancement?
Argument for psychedelic use	What are the arguments for psychedelic use identified within the source?
Arguments against psychedelic use	What are the arguments against psychedelic use identified within the source?
Main argument of the source	How does the source defend its main argument; what is the source’s stance?
Type of arguments employed in the source	What type of ethical reasoning/arguments used in the source? (i.e. utilitarian, deontological, etc.)
Other	“Other” denotes the possibility of creating new categories during the data charting process if needed.

We will not directly assess the quality of the included arguments. Although we plan to aggregate reasons into general groups—which can introduce biases ( Birchley & Ives, 2022)—we do so for the purpose of summary and organisation rather than evaluation. We also acknowledge that our categorization is inherently value-laden ( Birchley & Ives, 2022), and underscore that our argument collection and organisation is not absolute.

### Stage 5 - Data analysis and presentation

The forthcoming manuscript will provide readers with the completed scoping review of the published arguments for and against the use of psychedelics as moral bioenhancers. The results will be reported in accordance with PRISMA-ScR guidelines. We will include a PRISMA flow diagram detailing the results of our search methods and data extraction procedures. In addition, a summary table of the included works and their cited reasons will be provided. All members of the research team will be involved in crafting a cohesive cataloguing system to summarise the various arguments collected.

## Concluding remarks

Beyond moral bioenhancement, psychedelics may provide other forms of enhancement, such as cognitive or neurological enhancement more generally. We recognize that some discussions, including work by Dees (2007), already offer some forms of informal argument summary for and against the use of general neuroenhancers. Nevertheless, we are not aware of any discussion that specifically addresses *psychedelics* as *moral* bioenhancers. However, we ought to consider how psychedelics are distinct from other forms of neuroenhancers. Indeed, some suggest that psychedelics have a unique impact on user capacity ( Neitzke-Spruill *et al.*, 2024), creating issues that deserve special consideration. ^7^

As well, this scoping review’s extensive search of the literature bolsters our ability to comment on both common *and uncommon* arguments cited for and against the use of psychedelics as moral bioenhancers. Indeed, one of the most compelling benefits of a systematic review of reasons—or, in our case, a scoping review—relative to an informal review of reasons is the compiled reference list and "the table that summarises the position taken by each included publication" ( Sofaer & Strech, 2012, p. 326). Foreseeably, this information will shed light on areas that require further clarification while also helping policy-makers and academics navigate an emerging literature ( Sofaer & Strech, 2012).

## Ethics and consent

Ethical approval and consent were not required.

## Data Availability

No data are associated with this article.

## Software availability

Addressing our use of the proprietary Claude 3.5 Sonnet large language model, as discussed below, we note that other researchers could use any AI software with citation-formatting capacity in attempt to replicate our approach. Although Covidence is not an open access programme, we recommend a free alternative, Rayyan, if other teams are interested in following our methodology.
