# Supplementary material for: Psychedelics as moral bioenhancers: Protocol for a scoping review of ethical arguments for and against
Source: Wellcome Open Res. 2025 Jan 13;10:3. [Version 1] doi: 10.12688/wellcomeopenres.23414.1 (PMC11933787; doi:10.12688/wellcomeopenres.23414.1)
Supplement: Peer review history

# Reviewer report: Reviewer response for version 1

**DOI:** 10.21956/wellcomeopenres.25822.r120087

**Published:** 2025-03-24

**Authors:** Pei Hua Huang

**Affiliations:** 1 The University of Adelaide, Adelaide, South Australia, Australia

This is a very concise protocol for conducting a scoping review of ethical issues raised by the proposal for psychedelic moral bioenhancement. The authors provide a comprehensive introduction to moral bioenhancement, which in turn serves as a solid justification for (1) the importance of conducting a scoping review of psychedelic moral bioenhancement and (2) the methodology proposed by the authors. 

**I have three suggestions to the authors:**

 (1): This is really minor. In both the methodology and plain language summary, Philosopher's Index appears twice in the list of databases this research intends to use. I believe this is just a typo and can be fixed very easily.

 Also, I suggest the authors consider adding Douglas (2013) to provide a more comprehensive background for the fine tuning argument; Chan and Harris (2011), Huang and Tsu (2018) for the importance of moral flexibility.

 (2): On page 4, the authors mentioned that Earp (2018) proposes three desiderata for a moral bioenhancer "in response to such [aforementioned]  concerns." It is difficult for me to see how keeping 'bio' in moral bioenhancement addresses concerns such as those raised by Wiseman (2014; 2016). The biggest benefit of keeping "bio" in moral bioenhancement, I think, is to facilitate the discussion by specifying that the intervention under discussion must involve the use of biochemicals. The authors should revisit the passages and make it clearer what they'd like to achieve with the introduction to the three desirata. 

 (3): On page 5, the authors briefly introduced the effects of 'classic psychedelics' working via the (5-HT)2A receptor, which leaves readers with the impression that the review should focus on psychedelics working via the (5-HT)2A receptor. However, as the authors explained on page 6, the review should not limit itself to substances working primarily via the (5-HT)2A receptor, but should also include other hallucinogenic substances like ketamine (which is an NMDA receptor antagonist). Given that the authors aim to include substances that do not work primarily via  the (5-HT)2A receptor, I suggest the authors consider revising the passages on page 5 to enhance clarity.

Is the study design appropriate for the research question?

Yes

Is the rationale for, and objectives of, the study clearly described?

Yes

Are sufficient details of the methods provided to allow replication by others?

Yes

Are the datasets clearly presented in a useable and accessible format?

Yes

Reviewer Expertise:

Bioethics, Neuroethics, Philosophy of Medicine, Philosophy of Science

I confirm that I have read this submission and believe that I have an appropriate level of expertise to confirm that it is of an acceptable scientific standard.

## References

[1] : Moral enhancement via direct emotion modulation: a reply to John Harris. *Bioethics* .2013;27(3) : 10.1111/j.1467-8519.2011.01919.x 160-8 10.1111/j.1467-8519.2011.01919.x 22092503 PMC3378474

[2] : Moral enhancement and pro-social behaviour. *Journal of Medical Ethics* .2011;37(3) : 10.1136/jme.2010.041434 130-131 10.1136/jme.2010.041434 21325675

[3] Biomedical Moral Enhancement in the Face of Moral Particularism,Huang, P.-H., & Tsu, P. S.-H. *Royal Institute of Philosophy Supplements* .2018;83, 189-208.;

# Reviewer report: Reviewer response for version 1

**DOI:** 10.21956/wellcomeopenres.25822.r117550

**Published:** 2025-02-05

**Authors:** Naomi Scheinerman

**Affiliations:** 1 The Ohio State University, Columbus, Ohio, USA

More explicit identification of what a moral enhancement is and how psychedelics allow that would be helpful. There were indeed references to the "increased patients" and "opening the heart" and "increasing one's capacity to maintain perspective in response to strong emotional states" that seem more important in personality enhancement, but the connection to moral enhancement needed to be made stronger. How do these dispositions manifest in moral behavior?

Is the study design appropriate for the research question?

Yes

Is the rationale for, and objectives of, the study clearly described?

Yes

Are sufficient details of the methods provided to allow replication by others?

Yes

Are the datasets clearly presented in a useable and accessible format?

Yes

Reviewer Expertise:

Ethics, law, politics, regulation, and political thought.

I confirm that I have read this submission and believe that I have an appropriate level of expertise to confirm that it is of an acceptable scientific standard.
